# Comment on “Pretreatment Hepatitis C Virus NS5A/NS5B Resistance-Associated Substitutions in Genotype 1 Uruguayan Infected Patients”

**DOI:** 10.1155/2018/8698263

**Published:** 2018-11-05

**Authors:** Sana Eybpoosh, Mona Kiminezhad Malaie

**Affiliations:** ^1^Department of Epidemiology and Biostatistics, Research Centre for Emerging and Reemerging Infectious Diseases, Pasteur Institute of Iran, Tehran, Iran; ^2^HIV/STI Surveillance Research Center and WHO Collaborating Center for HIV Surveillance, Institute for Futures Studies in Health, Kerman University of Medical Sciences, Kerman, Iran

We very much enjoyed reading the recently published article by Aldunate et al. [1] in which the authors evaluated the presence of naturally occurring NS5A and NS5B resistance-associated substitutions (RASs) among drug-naïve patients who were chronically infected with Hepatitis C virus (HCV) genotype 1 in Montevideo, Uruguay. The frequency of targeted RASs in this study is estimated based on 31 HCV patients conveniently recruited from referees to a gastroenterology clinic in Montevideo, Uruguay. The authors found that naturally occurring NS5A and NS5B RASs were present in 8% and 19.2% of their sample, respectively. This finding is important as it is the first evidence for the presence of NS5A and NS5B RASs in Montevideo, Uruguay. However, we think that there are some points that should be considered about the results' validity and generalizability.

First, the authors have declared that the samples were selected among chronically infected HCV patients. These samples are then used for phylogenetic analysis to confirm the genotype of the isolated viruses and their genetic similarity and evolutionary relationships with viruses of other parts of the world. However, given the 31 samples collected in this study are selected from chronic HCV cases, they may have reached to partial or full “substitution (genetic) saturation.” Substitution saturation results from multiple substitutions occurring at single nucleotide sites over time [2]. It is well acknowledged that virus replication during the chronic phase of infection continuously drives a diverging and diversifying virus population through the act of “nucleotide substitution.” The longer the infection period, the more likelihood of each nucleotide site within the virus genome would occur [3–5]. *In vivo* studies have indicated that during the natural course of chronic HCV infection the dominant genetic mechanism acting on the HCV genome has been either continuous nucleotide substitutions or selective overgrowth of preexisting minor variants from the large spectrum of quasi-species populations [6]. Substitution saturation decreases the phylogenetic signal in the sequence alignment, adversely affecting the quality and accuracy of phylogenetic inferences. When full substitution saturation is reached, the query sequences are no longer informative about the underlying evolutionary processes, and hence, any estimate of genetic distances or phylogenetic trees, no matter which analytic method is used, will be meaningless. This happens because nucleotide sequences will tend to make cluster according to the degree of similarity in their base composition, irrespective of their true evolutionary relationships [2]. Aldunate et al. [1] have not tested the data for substitution saturation. If substitution saturation is substantial in their data, then the genotype assignments as well as the evolutionary relationships reconstructed by the authors might be incorrect. There are simple programs, such as DAMBE [7], that would have allowed the authors to assess if chronic infection with HCV has caused substantial substitution saturation in their data. Overlooking the problem of substitution saturation is common in the field. While saturation occurs more rapidly in fast-evolving pathogens [2], few studies have well addressed this issue in their phylogenetic analyses [8–10].

Second, the small number of HIV patients conveniently, but not randomly, sampled from one clinic of one city in Uruguay does not provide a representative sample of the total HCV population in Uruguay. This small and “nonrandom” sample cannot reflect the true picture of the HCV epidemic as well as the prevalence and diversity of RASs in this country. There are some parts in the article where the authors have (properly) interpreted their findings as the “presence” of NS5A and NS5B RASs in the sample. However, in most parts of the Discussion, their estimate of RAS frequency and diversity has been generalized to the entire country. For example, comparing their findings to those reported by studies in other countries, they have concluded that the prevalence of NS5A and NS5B RASs varies across South American countries. However, such comparisons are prone to bias as these estimates are not nationally representative. Due to the same reason, conclusions about RAS diversity within Uruguay and across South American countries would be misleading. Within the entire population of HCV-infected individuals, there might be subgroups/areas with higher or lower prevalence and diversity of RASs, depending on the transmission rate of the viruses containing RASs in each area. Unbiased estimates of RAS prevalence and diversity can be obtained from nationwide surveys possessing large and representative samples, obtained from probabilistic/semiprobabilistic sampling methods that are suitable for such hard-to-reach populations, *e.g*., chain-referral sampling [11, 12].

In conclusion, the finding of this study is important as it provides the evidence for the presence of naturally occurring NS5A and NS5B RASs in a sample of HCV patients in Montevideo, Uruguay. However, caution should be made while interpreting the findings of this study because these estimates are based on a small nonrandom sample of HCV patients in one city of this country and cannot be generalized to the entire HCV population in the country. Also, as participants were all chronic HCV patients, the isolated viruses might have reached partial or substantial substitution saturation which adversely affects the accuracy of the assigned HCV genotypes as well as the validity of reconstructed evolutionary relationships. Use of large and probabilistic/semiprobabilistic sampling methods is recommended to provide generalizable estimates on drug resistance prevalence and diversity. Testing for substitution saturation is also highly recommended prior to phylogenetic analysis of rapidly evolving sequences or sequences isolated from chronically infected patients.
